# Loss of connexin43 in murine Sertoli cells and its effect on blood-testis barrier formation and dynamics

**DOI:** 10.1371/journal.pone.0198100

**Published:** 2018-06-01

**Authors:** Julia Hollenbach, Klaus Jung, Joanna Noelke, Hagen Gasse, Christiane Pfarrer, Mirja Koy, Ralph Brehm

**Affiliations:** 1 Institute for Anatomy, University of Veterinary Medicine Hannover, Hannover, Germany; 2 Institute for Animal Breeding and Genetics, University of Veterinary Medicine Hannover, Hannover, Germany; 3 Institute for Immunology, University of Veterinary Medicine Hannover, Hannover, Germany; University Hospital of Münster, GERMANY

## Abstract

Connexin43 (Cx43) is the predominant testicular gap junction protein and in cases of impaired spermatogenesis, Cx43 expression has been shown to be altered in several mammals. Amongst other functions, Cx43 is supposed to regulate junction formation of the blood-testis barrier (BTB). The aim of the present study was to investigate the expression pattern of different tight junction (TJ) proteins of the murine BTB using SC-specific Cx43 knockout mice (*SCCx43KO*). Adult homozygous male *SCCx43KO* mice (*SCCx43KO*^*-/-*^) predominantly show an arrest of spermatogenesis and SC-only tubules that might have been caused by an altered BTB assembly, composition or regulation. TJ molecules claudin-3, -5 and -11 were examined in adult wild type (WT) and *SCCx43KO*^*-/-*^ mice using immunohistochemistry (IHC) and quantitative real-time PCR (qRT-PCR). In this context, investigation of single tubules with residual spermatogenesis in *SCCx43KO*^*-/-*^ mice was particularly interesting to identify a potential Cx43-independent influence of germ cells (GC) on BTB composition and dynamics. In tubules without residual spermatogenesis, a diffuse cytoplasmic distribution pattern for claudin-11 protein could be demonstrated in mutant mice. Nevertheless, claudin-11 seems to form functional TJ. Claudin-3 and -5 could not be detected immunohistochemically in the seminiferous epithelium of those tubules. Correspondingly, claudin-3 and -5 mRNA expression was decreased, providing evidence of generally impaired BTB dynamics in adult KO mice. Observations of tubules with residual spermatogenesis suggested a Cx43-independent regulation of TJ proteins by GC populations. To determine initial BTB formation in peripubertal *SCCx43KO*^*-/-*^ mice, immunohistochemical staining and qRT-PCR of claudin-11 were carried out in adolescent *SCCx43KO*^*-/-*^ and WT mice. Additionally, BTB integrity was functionally analysed using a hypertonic glucose fixative. These analyses revealed that *SCCx43KO*^*-/-*^ mice formed an intact BTB during puberty in the same time period as WT mice, which however seemed to be accelerated.

## Introduction

Connexin43 (Cx43) is the predominant testicular gap junction (GJ) protein. Within the seminiferous epithelium, it connects adjacent Sertoli cells (SC) as well as SC and germ cells (GC). Cx43 influences normal testis development and is necessary for initiation and physiological progression of spermatogenesis [1–10]. In tubules with normal spermatogenesis, its distribution pattern seems to be similar in men and several animal species [2, 11–17]. In cases of impaired spermatogenesis, Cx43 expression changes can therefore be associated with infertility [11, 13, 17–22]. Furthermore, Cx43 is supposed to play a role in blood-testis barrier (BTB) formation, regulation and dynamics [9, 23]. The BTB is built up during puberty in the basal third of the seminiferous epithelium and divides this epithelium into a basal and an adluminal compartment. The basal compartment contains diploid GC (spermatogonia and preleptotene primary spermatocytes), whereas the adluminal compartment contains more advanced and haploid GC (late primary spermatocytes, secondary spermatocytes, round and elongated spermatids) [24–27]. A functional BTB is crucial for GC survival and normal progression of spermatogenesis. However, the BTB is not rigid but represents a very dynamic structure as preleptotene/leptotene GC must cross the BTB to reach the adluminal compartment without compromising the integrity of this barrier. This occurs during stages VIII-IX of the murine seminiferous epithelial cycle. In addition to GJ, other junctional proteins, like adherens junctions (AJ) and tight junctions (TJ), are mainly involved in BTB establishment [28–32]. However, the BTB is more than just the junctions as this anatomical (physical) component has to interact with physiological and immunological components to create a barrier of maximal competence [33, 34].

Amongst others, TJ proteins occludin and claudin-3, -5 and -11 are important known components of the murine BTB [35–39]. Claudin-11 (also known as oligodendrocyte specific protein (OSP)) is expressed in SC during all stages of the murine seminiferous epithelial cycle and its knockout (KO) leads to morphological alterations in the testis from day 20 post partum (p.p.) onwards [37, 38, 40–42]. In contrast, a KO of occludin does not affect testicular morphology and function within the first 40 weeks p.p. in mice [43]. These observations suggest that claudin-11 possibly plays a key role in the initial forming of the BTB.

In mice, claudin-3 and -5 show a stage-specific expression pattern with highest expression levels in stages VIII-IX of the seminiferous epithelium. Both claudins can therefore be associated with BTB dynamics [38, 39, 44, 45]. Unlike claudin-11, these claudins are not solely expressed in SC but also in GC (claudin-3: preleptotene/leptotene spermatocytes; claudin-5: spermatogonia, spermatocytes and spermatids) [38, 39, 46–49]. Claudin-5 is also highly expressed in endothelial cells in the testis [39].

As a global KO of Cx43 leads to cardiac malformation and perinatal death [50], a SC specific Cx43 KO (*SCCx43KO*) mouse line, lacking Cx43 only in SC, was established using the Cre/LoxP recombination system. Homozygous male *SCCx43KO* mice (*SCCx43KO*^*-/-*^) are infertile with tubules showing an SC only syndrome (SCO) and/or an arrest of spermatogenesis at the level of spermatogonia, intratubular SC clusters and SC cytoplasmic vacuoles [5, 6]. Interestingly, some tubules show advanced GC development (spermatocytes or even spermatids). These tubules account for around 5% and a deficient or incomplete deletion of the Cx43 gene seems unlikely to explain residual spermatogenic activity as the efficiency of the Cre activity in the *AMH-Cre* mouse line is considered robust and highly cell specific [5, 51, 52]. *SCCx43KO*^*-/-*^ mice show signs of an insufficient maturation of SC [6, 7]. This is, amongst others, characterised by an altered immuno expression of the Androgen Receptor (AR) and dependent on the degree of the spermatogenic disorder [53].

As mentioned above, Cx43 is supposed to play a key role in BTB formation and dynamics. Unexpectedly, functional investigations revealed an intact but altered BTB in adult *SCCx43KO*^*-/-*^ mice [23]. Different junctional proteins (e.g. N-Cadherin, β-Catenin, occludin and claudin-11) are upregulated in *SCCx43KO*^*-/-*^ mice and ultrastructural examination via electron microscopy (but not immunoelectron microscopy) showed increased AJ and TJ per unit length of the BTB as compared to WT mice [9, 23]. These observations may lead to the conclusion that Cx43 is not crucial for establishing an intact BTB, but perhaps influences BTB dynamics. Thus, spermatogenic disorders in adult *SCCx43KO*^*-/-*^ mice might have been caused by an altered BTB assembly and/or disassembly, composition or regulation.

Therefore, in the first part of the present study, the expression pattern of the TJ proteins claudin-3, -5 and -11 was investigated in adult *SCCx43KO*^*-/-*^ mice and compared to WT mice, using immunohistochemistry (IHC) as well as quantitative real-time PCR (qRT-PCR). In the second part of the study, the initial formation of the BTB was investigated in peripubertal *SCCx43KO*^*-/-*^ mice. For this purpose, IHC and qRT-PCR of claudin-11 were carried out. Finally, functional investigations were performed to determine the integrity of the BTB in these mice.

## Material and methods

### Animals and tissue sampling

All animal experiments were approved by the Animal Rights Committee of the Regional Commission of Giessen (decision V54–19 c 20/15 c GI 18/1) and by the Lower Saxony State Office for Consumer Protection and Food Safety (reference numbers 33.9-425-05-11A120 and 33.9 42502-04-12/0877). The breeding strategy of *SCCx43KO*^*-/-*^ mice as well as genotyping PCR and confirmation of Cx43 gene loss are described elsewhere [5]. The mice were housed with 12 h light and 12 h dark cycles at a temperature between 20 and 24°C. Food and water were given ad libitum. Genotyped *SCCx43KO*^*-/-*^ and WT mice were anaesthetised by CO_2_ and killed by cervical dislocation. Both testes were removed immediately and either fixed in the respective fixative (immunohistochemistry (IHC) and functional investigation) or snap frozen in liquid nitrogen and stored at -80 °C (RNA extraction).

### Immunohistochemistry

For IHC, testes were fixed in Bouin’s solution for 48 h and subsequently transferred to 70% ethanol. Samples were embedded in paraffin wax using standard techniques and 4-μm sections were cut and mounted on glass slides (Histobond; Paul Marienfeld, Laboratory Glassware, Lauda-Königshofen, Germany). During deparaffinisation and rehydration, endogenous peroxidase was blocked using hydrogen peroxide.

All antibodies used in this study are summarised in Table 1. Same protocols were used for the negative controls except for the omission of the primary antibody. The isotype control was performed by substituting the primary antibody with a polyclonal anti-rabbit IgG antibody (Sigma Aldrich, München, Germany).

**Table 1:** Antibodies used for immunohistochemistry.

Protein	Primary antibody	Host Mono/Poly	Dilution	Secondary antibody
Beta-galactosidase	Anti-beta Galactosidase antibody (Abcam, ab616)	E. coli polyclonal	1:3000	Labelled Polymer-HRP Anti-rabbit, ready to use
Cx43	Connexin43 antibody (Invitrogen 71–0700)	Rabbit, polyclonal	1:250	Labelled Polymer-HRP Anti-rabbit, ready to use
Claudin-3	Anti-Claudin-3 (Invitrogen, 34–1700)	Rabbit, polyclonal	1:500	Biotinylated Goat Anti-Rabbit, 1:200
Claudin-5	Anti-Claudin-5 (Invitrogen, 34–1600)	Rabbit, polyclonal	1:1000	Biotinylated Goat Anti-Rabbit, 1:200
Claudin-11	Anti-Oligodendrocyte Specific Protein antibody (Abcam, ab53041)	Rabbit, polyclonal	1:2000	Labelled Polymer-HRP Anti-rabbit, ready to use

#### Beta-galactosidase and Cx43 (Confirmation of successful Cx43 gene deletion and Cx43 protein loss)

Beta-galactosidase and Cx43 IHC were performed with minor changes as previously described [5] to confirm successful Cx43 gene deletion and absence of Cx43 protein in *SCCx43KO*^*-/-*^ tubules with residual spermatogenesis. For Cx43 protein, antigen retrieval was conducted by boiling slides in sodium citrate buffer (pH 6.0) for 20 min at 96–99°C. For beta-galactosidase, no antigen retrieval was necessary and afterwards all sections were blocked using 3% bovine serum albumin for 20 min. Both primary antibodies were diluted in 1% BSA in PBS and incubated overnight at 4°C in a humidified chamber: beta-galactosidase (Abcam ab616, 1:3000), Cx43 (Invitrogen 71–0700, 1:250). On the following day, sections were incubated for 45 min with horseradish peroxidase (HRP)-conjugated secondary antibody (anti-rabbit EnVision; DAKO, Hamburg, Germany, ready to use) and immunoreaction was visualised by diaminobenzidin (DAB; EnVision, Dako, Hamburg, Germany). Sections were counterstained with hematoxylin, dehydrated and mounted with Eukitt^®^ (O. Kindler GmbH, Freiburg, Germany).

#### Claudin-3, -5 and -11

IHC was performed for claudin-3, -5 and -11 in adult WT and *SCCx43KO*^*-/-*^ mice (aged ≥ 60 days; n = 3 WT mice, 5 *SCCx43KO*^*-/-*^ mice with (n = 3) and without (n = 2) residual spermatogenesis), claudin-11 IHC was also performed in adolescent WT and *SCCx43KO*^*-/-*^ mice (aged 2, 8, 12, 14, 16 and 23 days; n = 2 mice/genotype and age group). For all three proteins, antigen retrieval was conducted by boiling slides in sodium citrate buffer (pH 6.0) for 20 min at 96–99°C. Sections were blocked using 3% bovine serum albumin for claudin-11 or 20% normal goat serum containing avidin blocking solution (Vector Laboratories, Burlingame, USA) for claudin-3 und -5 for 20 min. Incubation with primary antibodies was conducted overnight at 4°C in a humidified chamber. Primary antibodies were diluted in 1% BSA in PBS for claudin-11 (abcam 53041, 1:2000) and in 1% BSA in PBS containing biotin blocking solution (Vector Laboratories, Burlingame, USA) for claudin-3 (Invitrogen 34–1700, 1:500) and claudin-5 (Invitrogen 34–1600, 1:1000). On the following day, sections were incubated for 45 min with compatible secondary antibodies: horseradish peroxidase (HRP)-conjugated secondary antibody (anti-rabbit EnVision; DAKO, Hamburg, Germany, ready to use) for claudin-11 and biotinylated goat anti-rabbit secondary antibody (Vector Laboratories, Burlingame, USA, 1:200) for claudin-3 und -5. Claudin-3 and -5 sections were then incubated with avidin-biotin-peroxidase complex (Vectastain Elite ABC standard kit, Vector laboratories) for 30 min, followed by biotinylated tyramine for 15 min and again avidin-biotin-peroxidase complex for 15 min. Immunoreactivity was visualised by DAB (EnVision, Dako, Hamburg, Germany) and sections were counterstained with hematoxylin, dehydrated and mounted with Eukitt^®^ (O. Kindler GmbH, Freiburg, Germany).

### Functional investigation

**Fig 1:**
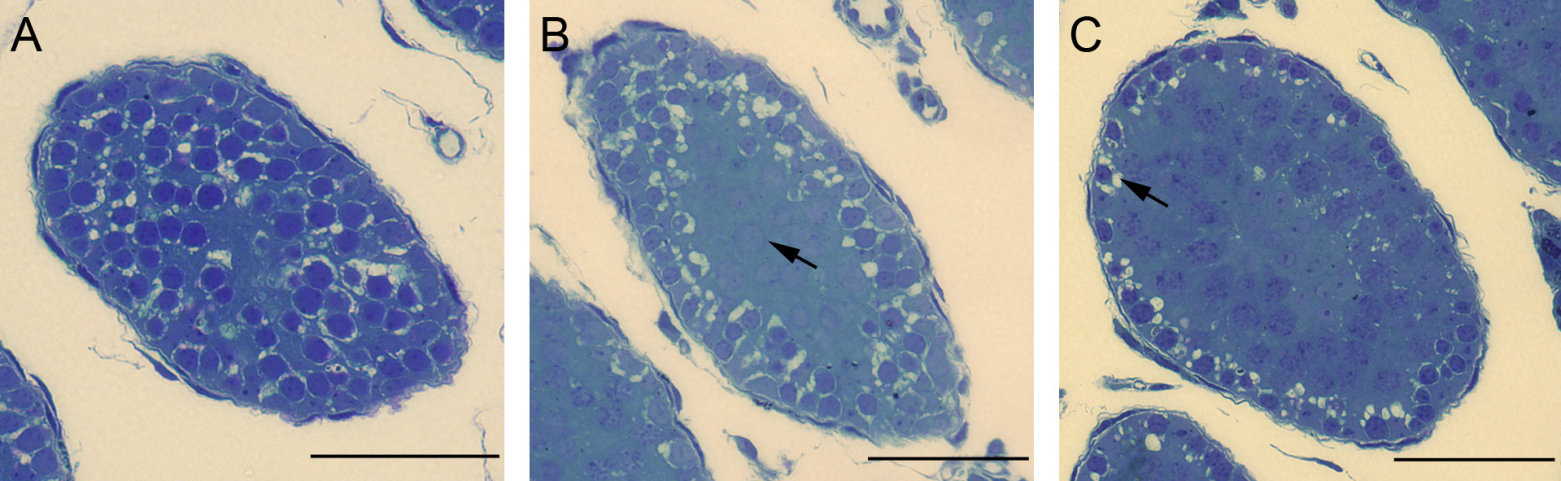
Classification of seminiferous tubules for evaluating the functional investigation. (A) In the category “open”, all tubular cells exhibit shrinkage artefact. (B) In the category “intermediate”, centrally located cells are already intact (arrow) and (C) in the category “closed” cell shrinkage is limited to the basal compartment of the seminiferous epithelium (arrow). Scale bars: 50 μm.

To investigate initial BTB formation in peripubertal *SCCx43KO*^*-/-*^ and WT mice (aged 10, 12, 14, 16 and 23 days), functional investigations were performed. Testes were fixed in a fixation solution containing 2% glutaraldehyde and 2% formaldehyde in 0.1 M sodium cacodylat buffer adjusted to pH 7.3 [54]. To determine BTB integrity, right testes were fixed in this solution additionally containing 10% glucose for two hours and subsequently fixed in the same fixative without glucose. Left testes were fixed without glucose and served as negative controls. All samples were fixed for at least 24 h at 4°C. Afterwards, the samples were dissected and rinsed in 0.1 M sodium cacodylat buffer for three hours. Tissue blocks were post fixed in 1% osmium tetroxide for two hours and subsequently rinsed in 0.1 M sodium cacodylat for 20 min. After dehydration, samples were contrasted with uranyl acetate and embedded in Epon 812 (Serva Electrophoresis GmbH, Heidelberg, Germany). Semithin sections were stained with toluidine blue and mounted with DePex (Serva Electrophoresis GmbH, Heidelberg, Germany). For evaluation, seminiferous tubules were classified into three categories (Table 2 and Fig 1) according to Willems et al. [55]. Between 244 and 346 tubules were classified for each age group and genotype distributed to at least two mice per genotype and the percentage of each category was calculated.

**Table 2:** Classification of seminiferous tubules for evaluating the functional investigation.

Category	Morphology	Meaning
Open	Cell shrinkage in all tubular cells	No functional TJ
Intermediate	Cell shrinkage over several cell layers, centrally located cells are intact	Functional TJ, which hadn’t arranged consistently in the basal third of the seminiferous epithelium
Closed	Cell shrinkage is restricted to the lowest cell layer (basal compartment)	Intact BTB

### RNA extraction and cDNA synthesis

For adult mice, total RNA of whole testes homogenates (n = 3 mice/genotype) was prepared using PureLink^®^RNA Mini Kit (Thermo Fisher Scientific Inc., Waltham, USA) in accordance with the manufacturer’s protocol. All animals used in these experiments showed no signs of residual spermatogenesis. RNA was quantified by using spectrophotometric determination of the optical density at 260 nm (DeNovix^®^ DS-11, DeNovix Inc., Wilmington, USA). DNase digestion was performed using DNase I recombinant, RNase-free (Roche, Mannheim, Germany), followed by reverse transcription of 1.0 μg total RNA in a 20 μl reaction volume using GoScript Reverse Transcriptase Kit (Promega GmbH, Mannheim, Germany) in accordance with the manufacturer’s protocol. Due to low testis weight of adolescent mice (aged 2, 8, 10, 12 and 14 days p.p.), total RNA (n = 3 mice/genotype per age group) was extracted using RNeasy Plus Micro Kit (Qiagen, Hilden, Germany) as recommended by the manufacturer. RNA quantification and reverse transcription were conducted as described above.

### Quantitative real-time PCR

Before being used in qRT-PCR, all primers (listed in Table 3) were tested in normal PCR using standard protocol to confirm their specificity. As primers for the housekeeping genes (*Hsp90ab1* and *Actb*) were self-designed (PubMed, Primer designing tool), their PCR products were isolated using MinElute PCR Purification Kit (Qiagen, Hilden, Germany) and sequenced at SEQLAB Sequence Laboratories (Goettingen, Germany).

**Table 3:** Primers used in this study.

Gene	Primersequence, forward	Used primer volume	Product length	Reference
	Primersequence, reverse			
*Hsp90ab1*	TGCTAAGTCTGGCACGAAGG	1.5 μl	166 bp	Pubmed
	AGACGACTCCCAGGCATACT	4.5 μl		
*Actb*	CACTGTCGAGTCGCGTCC	1.5 μl	102 bp	Pubmed
	CGCAGCGATATCGTCATCCA	4.5 μl		
*Claudin-3*	AACTGCGTACAAGACGAGACG	1.5 μl	143 bp	[56]
	GGCACCAACGGGTTATAGAAAT	0.5 μl		
*Claudin-5*	GGCGATTACGACAAGAAGAACT	1.5 μl	193 bp	[57]
	TAGTGATGGTCAACGGACTCTG	4.5 μl		
*Claudin-11*	CGTCATGGCCACTGGTCTCT	1.5 μl	82 bp	[8]
	GGCTCTACAAGCCTGCACGTA	1.5 μl		

Quantitative Real-Time PCR was performed using SYBR^®^ Green PCR Master Mix and Step One Plus Real-Time Cycler (Applied Biosystems, Waltham, USA) under the following cycling conditions: 10 min at 95°C, 40 cycles of denaturation at 95°C for 15 s, annealing and extension at 60°C for 60 s with fluorescence detection during the annealing/extension step. Due to the unspecific dsDNA binding property of the SYBR^®^ Green dye, melting curve analysis was performed to confirm PCR product purity. To calculate the relative gene expression of claudin-3, -5 and -11, values were normalised to the amount of two housekeeping genes (*Hsp90ab1* and *Actb*) by the ΔΔC(t) method. The quantitative proportion between forward and reverse primer (concentration 5 μM) was optimised for each primer pair summarised in Table 3. Primer amplification efficiencies were analysed by using the standard curve method with various dilution steps (1:2, 1:4, 1:8, 1:16 for claudin-5, 1:10, 1:100, 1:1.000, 1:10.000 for claudin-3, -11, Hsp90ab1 and Actb). All reactions were run in triplicate with a 25-μl reaction volume containing 12.5 μl SYBR^®^ Green PCR Master Mix and 1 μl cDNA (undiluted for claudin-5, 1:10 dilution for claudin-3, -5, Hsp90ab1 and Actb). After adding the corresponding amount of primers, the volume was filled up with nuclease-free water. For each primer pair, two negative controls were implemented containing nuclease-free water instead of cDNA. Evaluation was carried out using Step One^™^ 2.3 Software (Applied Biosystems, Waltham, USA).

### Data analysis

Expression fold changes between KO and WT samples were calculated using the ΔΔC(t) method [58, 59], i.e. the fold change (KO/WT) was defined by 2^-ΔΔ(KO|WT)^. For down-regulated targets, fold changes were also described by negative reciprocal, i.e. -1/2^-ΔΔ(KO|WT)^ as proposed in the protocols by Schmittgen and Livak [60].

In order to derive 95%-confidence intervals for the fold changes, the following procedure was used: Denoting the mean CT values for KO and WT in reference and target samples by M_KO|REF_, M_KO|TAR_, M_WT|REF_, M_WT|TAR_, respectively, and related variances by V_KO|REF_, V_KO|TAR_, V_WT|REF_, V_WT|TAR_. Since in our case, measurements in the reference and the target are paired, we can also derive the covariances COV_KO_ = COV(KO_REF_, KO_TAR_) and COV_WT_ = COV(WT_REF_, WT_TAR_). The mean difference between reference and target in the KO and WT group are then given by

1. Δ_*KO*_ = *M*_*KO*|*TAR*_ − *M*_*KO*|*REF*_ and Δ_*WT*_ = *M*_*WT*|*TAR*_ − *M*_*WT*|*REF*_, respectively. Using statistical theory, the variances of Δ_KO_ and Δ_WT_ are given by
2. *V*_Δ(*KO*)_ = *V*_*KO*|*REF*_ + *V*_*KO*|*TAR*_ − *COV*_*KO*_ and *V*_Δ(*WT*)_ = *V*_*WT*|*REF*_ + *V*_*WT*|*TAR*_ − *COV*_*WT*_ respectively. Since measurements in the KO and WT group are uncorrelated, the mean difference between KO and WT group and the related variance are given by
3. ΔΔ_*KO*|*WT*_ = Δ_*KO*_ − Δ_*WT*_ and V_ΔΔ(*KO*|*WT*)_ + *V*_*WT|REF*_ Assuming a normal distribution and using the quantities in (3), lower (L) and upper (U) bounds of the confidence intervals can be derived by:
4. $L=\Delta \Delta_{KO|WT}-z_{1-\alpha /2}\cdot \sqrt{V_{\Delta \Delta (KO|WT)}}$ and $\text{U}=\Delta \Delta_{KO|WT}+z_{1-\alpha /2}\cdot \sqrt{V_{\Delta \Delta (KO|WT)}}$,
  where z_1-α/2_ denotes the (1-α/2)-quantile of the normal distribution. Changing to the scale of the final fold change, the final bounds are given by L’ = 2^-L^ and U’ = 2^-U^.

In addition, t-tests were performed to test the null hypothesis that the fold change is equal to 1 versus the alternative that the fold changes are significantly different from 1. All analyses were performed using the software R (V3.2.3, www.r-project.org).

## Results

### Confirmation of successful Cx43 gene deletion and Cx43 protein loss in tubules with residual spermatogenesis

#### Beta-galactosidase IHC

**Fig 2:**
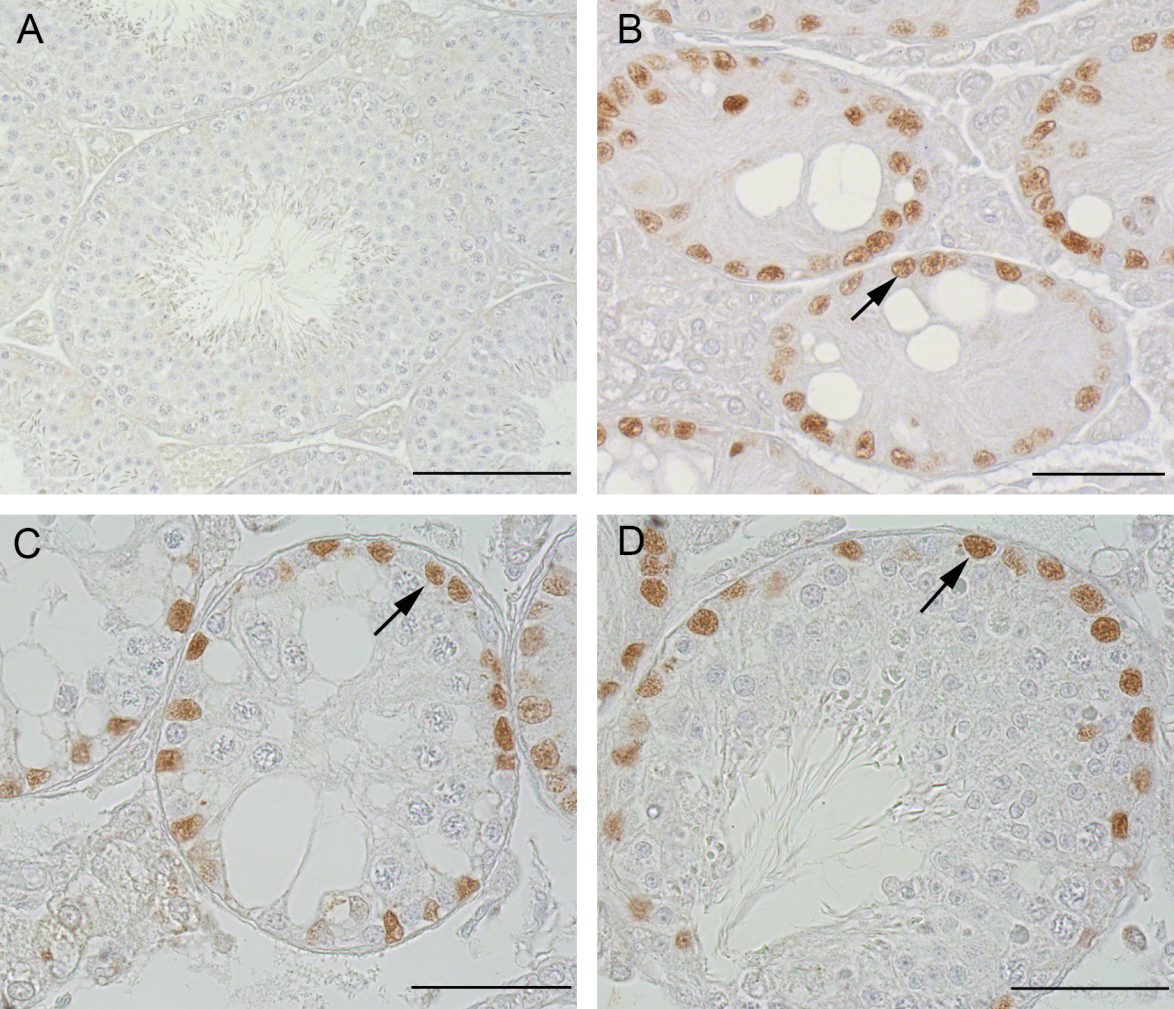
Beta-galactosidase immunohistochemistry in adult WT and *SCCx43KO*^*-/-*^ mice. (A) WT tubules not showing any immunoreaction for beta-galactosidase. Scale bar: 100 μm. (B) *SCCx43KO*^*-/ -*^tubules without residual spermatogenesis showing evident immunoreaction in SC nuclei (arrow). Scale bar: 50 μm. (C and D) *SCCx43KO*^*-/-*^ tubules with residual spermatogenesis also showing evident immunoreaction in SC nuclei (arrows), which confirms the successful deletion of the Cx43 gene. Scale bars: 50 μm.

To confirm successful deletion of the Cx43 gene in SC of *SCCx43KO*^*-/-*^ mice with residual spermatogenesis, beta-galactosidase IHC was performed [5]. SC nuclei in all *SCCx43KO*^*-/-*^ tubules, with and without residual spermatogenesis, showed immunopositive reactions, confirming successful gene deletion. All other cells were negative (Fig 2B–2D). SC nuclei of WT tubules were immunonegative (Fig 2A).

#### Cx43 IHC

**Fig 3:**
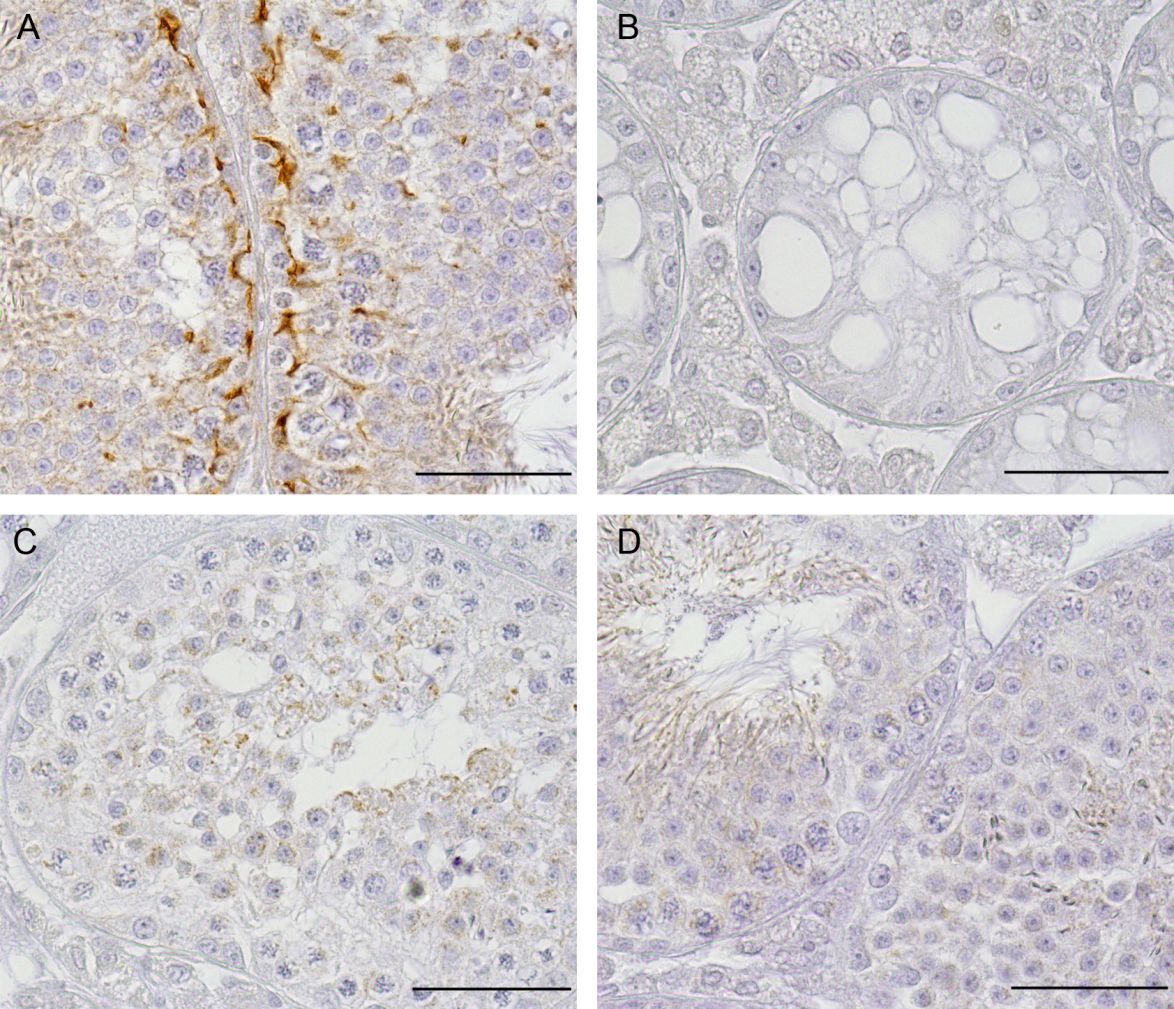
Cx43 immunohistochemistry. (A) WT tubules showing an immunopositive reaction in the area of the BTB. (B) *SCCx43KO*^*-/-*^ tubules showing an SCO syndrome. No immunoreaction was visible in these tubules. (C and D) *SCCx43KO*^*-/-*^ tubules with residual spermatogenesis not showing any immunopositive reaction in the area of the BTB. Scale bars: 50 μm.

To confirm loss of Cx43 protein in tubules of *SCCx43KO*^*-/-*^ mice with residual spermatogenesis, Cx43 IHC was performed. No immunoreaction was detectable in any tubules of adult mutant mice (Fig 3B–3D).

### Expression of the TJ proteins claudin-3, -5 and -11 in adult *SCCx43KO*^*-/-*^ mice

#### Claudin-3 IHC

**Fig 4:**
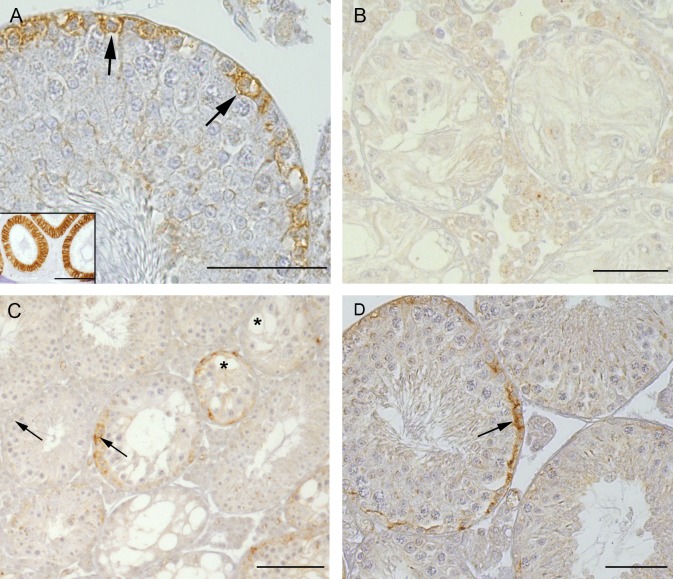
Claudin-3 immunohistochemistry in adult WT and *SCCx43KO*^*-/-*^ mice. (A) WT tubule showing an immunopositive reaction in the basal part at stage VIII of the seminiferous epithelium (arrows). Scale bar: 50 μm. The positive immunoreaction in the epididymis confirms the binding specificity of the antibody (A, inset). (B) *SCCx43KO*^*-/-*^ tubules with SCO not showing any immunopositive reaction. Scale bar: 50 μm. (C) *SCCx43KO*^*-/-*^ tubules with residual spermatogenesis containing spermatocytes and round spermatids showing a heterogeneous staining pattern with immunopositive and negative reactions in the basal part, apparently independent from the containing GC population. The asterisks indicate two tubules containing spermatocytes. The left one shows an immunopositive reaction for claudin-3 at the basal part of the seminiferous epithelium, the right one is immunonegative. The arrows indicate an immunopositive (right) and an immunonegative (left) reaction in tubules containing round spermatids. Scale bar: 100 μm. (D) *SCCx43KO*^*-/-*^ tubules with qualitative normal spermatogenesis showing the same stage dependent staining pattern as adult WT mice with an immunopositive reaction at stage VIII of the seminiferous epithelium (arrow). Scale bar: 50 μm.

As previously described [38, 45–47], also in the present study an immunopositive reaction for claudin-3 could be seen in the basal part of the seminiferous epithelium at stage VIII in adult WT mice (Fig 4A). As claudin-3 is also highly expressed in the epididymal epithelium [61], the positive immunoreaction in the epididymis served as positive control for the specific binding property of the antibody (Fig 4A inset). In *SCCx43KO*^*-/-*^ tubules without residual spermatogenesis (SCO or spermatogonial arrest), no claudin-3 protein was detectable (Fig 4B). In tubules with residual spermatogenesis containing spermatocytes and round spermatids, claudin-3 immunoreaction showed a heterogeneous staining pattern with immunopositive and negative reactions in the basal part of the seminiferous epithelium, which was apparently independent from the containing GC population. There were tubules containing spermatocytes showing a positive immunoreaction and some showing no immunoreaction (Fig 4C, asterisks). This staining pattern was similar in tubules containing round spermatids (Fig 4C, arrows). In tubules with qualitatively normal spermatogenesis, the immunostaining pattern for claudin-3 was similar to that of adult WT mice with an immunopositive reaction in the basal part of the seminiferous epithelium at stage VIII (Fig 4D).

#### Claudin-5 IHC

**Fig 5:**
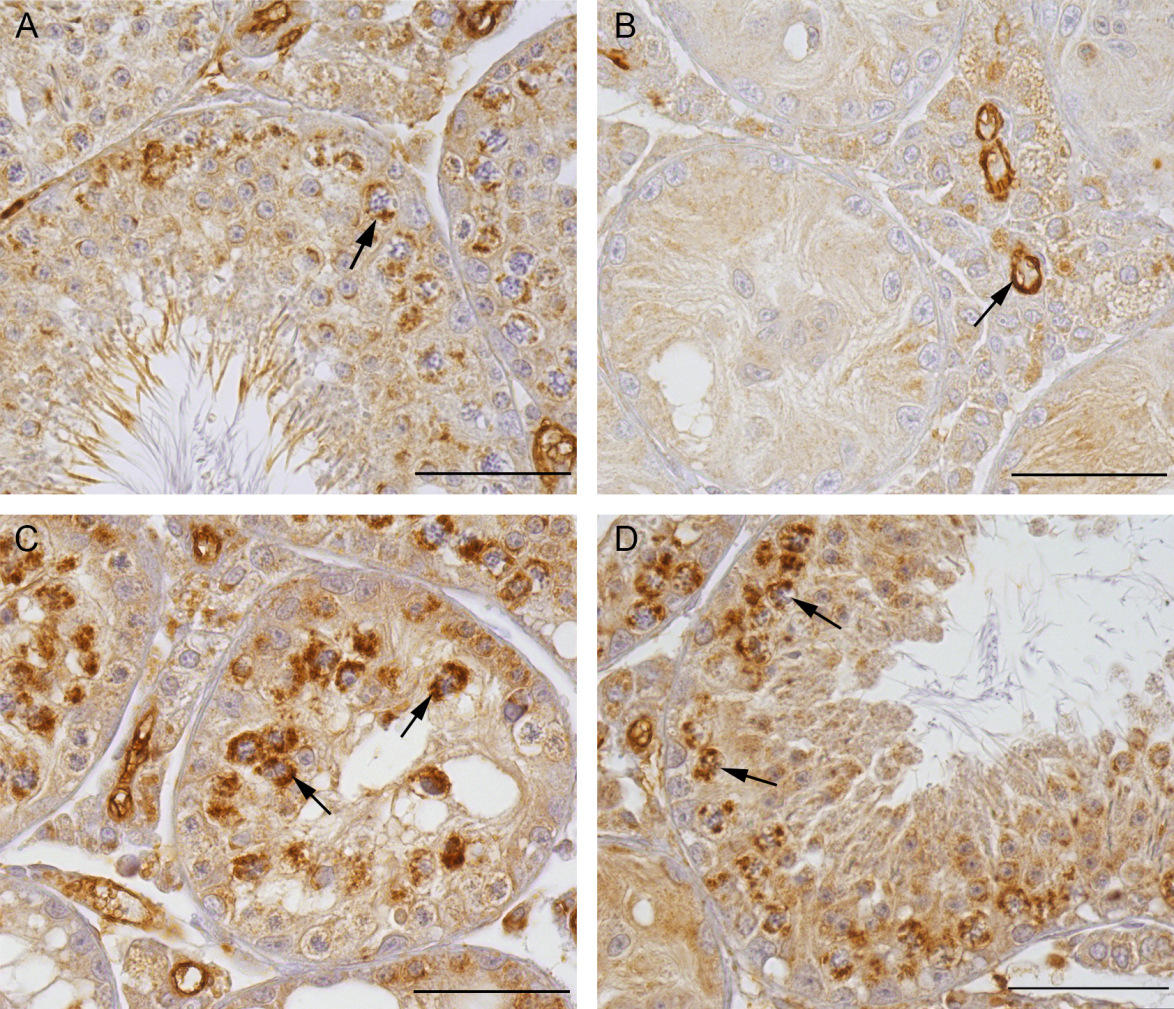
Claudin-5 immunohistochemistry in adult WT and *SCCx43KO*^*-/-*^ mice. (A) WT tubule showing an immunopositive reaction around spermatocytes in the basal part at stage VIII of the seminiferous epithelium (arrow). Scale bar: 50 μm. (B) *SCCx43KO*^*-/-*^ tubule with SCO not showing any immunopositive reaction. Endothelial cells show an immunopositive reaction (arrow), confirming the binding specificity of the antibody. Scale bar: 50 μm. (C) *SCCx43KO*^*-/-*^ tubules with residual spermatogenesis showing an immunopositive reaction around spermatocytes (arrows). Scale bar: 50 μm. (D) *SCCx43KO*^*-/-*^ tubule with qualitative normal spermatogenesis showing the same staining pattern as adult WT mice (arrows). Scale bar: 50 μm.

Like claudin-3, in the present study claudin-5 also showed an immunopositive reaction in the basal part of the seminiferous tubules at stage VIII of the seminiferous epithelium cycle in adult WT mice (Fig 5A) as previously described [39]. In *SCCx43KO*^*-/-*^ tubules without residual spermatogenesis (SCO or spermatogonial arrest), no claudin-5 protein was detectable (Fig 5B), but an immunopositive reaction around spermatocytes was visible in tubules with residual spermatogenesis (Fig 5C). In tubules with qualitatively normal spermatogenesis, the staining pattern for claudin-5 was similar to that of adult WT mice (Fig 5D). As claudin-5 is also highly expressed in endothelial cells [39], the positive reactions of the blood vessels in both genotypes served as a positive control for the specific binding property of the antibody (Fig 5A–5D).

#### Claudin-11 IHC

**Fig 6:**
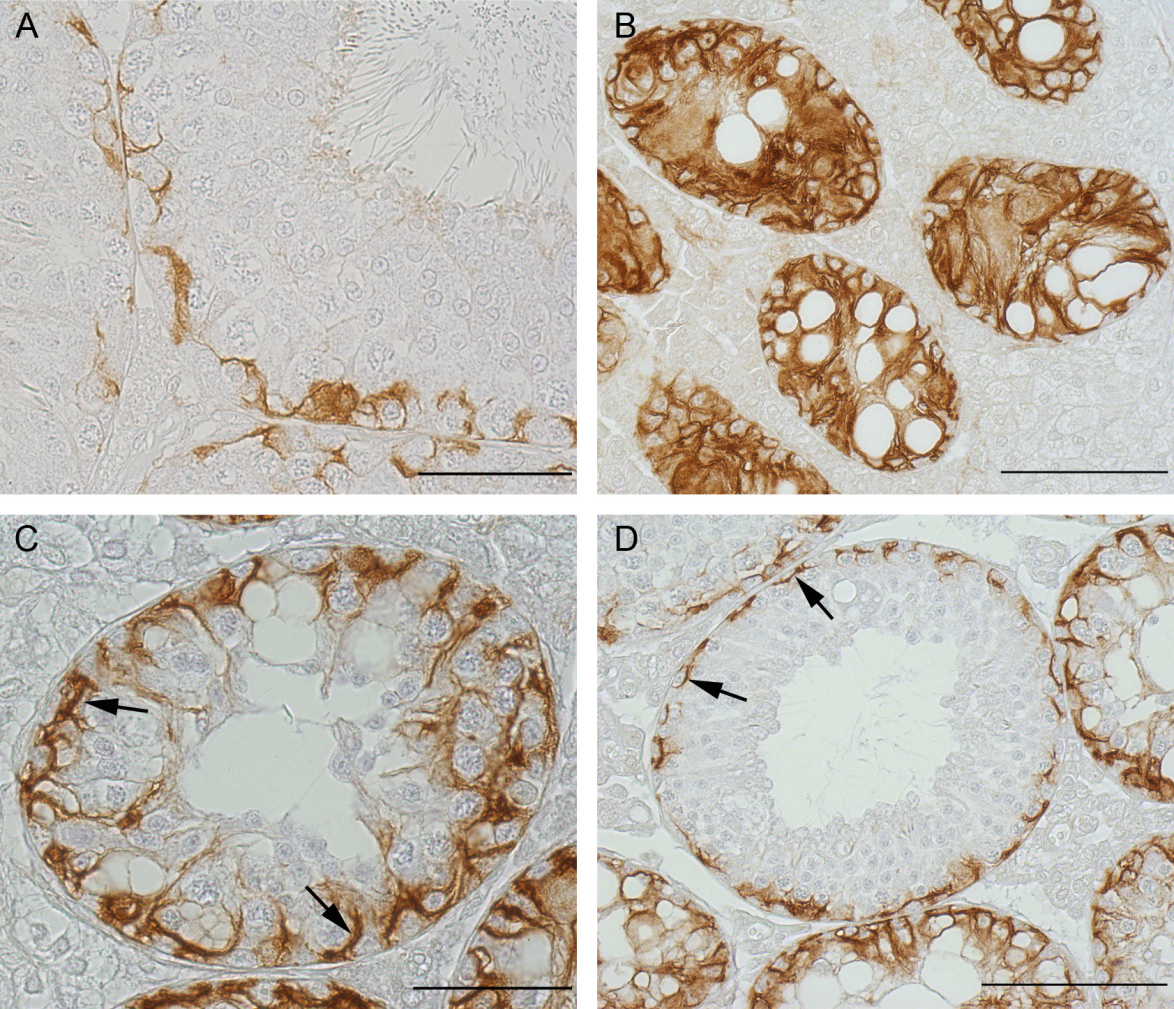
Claudin-11 immunohistochemistry in adult WT and *SCCx43KO*^*-/-*^ mice. (A) WT tubule showing a fine linear immunopositive reaction in the basal part of the seminiferous epithelium. Scale bar: 50 μm. (B) *SCCx43KO*^*-/-*^ tubules with SCO showing an apparently increased immunoreaction and a diffuse cytoplasmic distribution pattern. Scale bar: 100 μm. (C) *SCCx43KO*^*-/-*^ tubule containing round spermatids showing a finer staining pattern and localisation towards the BTB (arrows). Scale bar: 50 μm. (D) *SCCx43KO*^*-/-*^ tubule with qualitative normal spermatogenesis showing the same staining pattern as adult WT mice (arrows). Scale bar: 100 μm.

As previously observed by others [37, 38, 45], in the present study claudin-11 showed a fine linear staining pattern in the basal third of the seminiferous epithelium throughout all stages of the seminiferous epithelial cycle in adult WT mice (Fig 6A). In *SCCx43KO*^*-/-*^ tubules without residual spermatogenesis, an apparently increased immunoreaction as well as a diffuse cytoplasmic distribution pattern for claudin-11 could be demonstrated for the first time in SC (Fig 6B). In tubules with residual spermatogenesis containing round spermatids, claudin-11 showed a finer staining pattern and a localisation of the immunostaining towards the BTB (Fig 6C). In tubules with qualitative normal spermatogenesis, the staining pattern for claudin-11 was similar to that of adult WT mice (Fig 6D).

#### Quantitative real-time PCR

**Fig 7:**
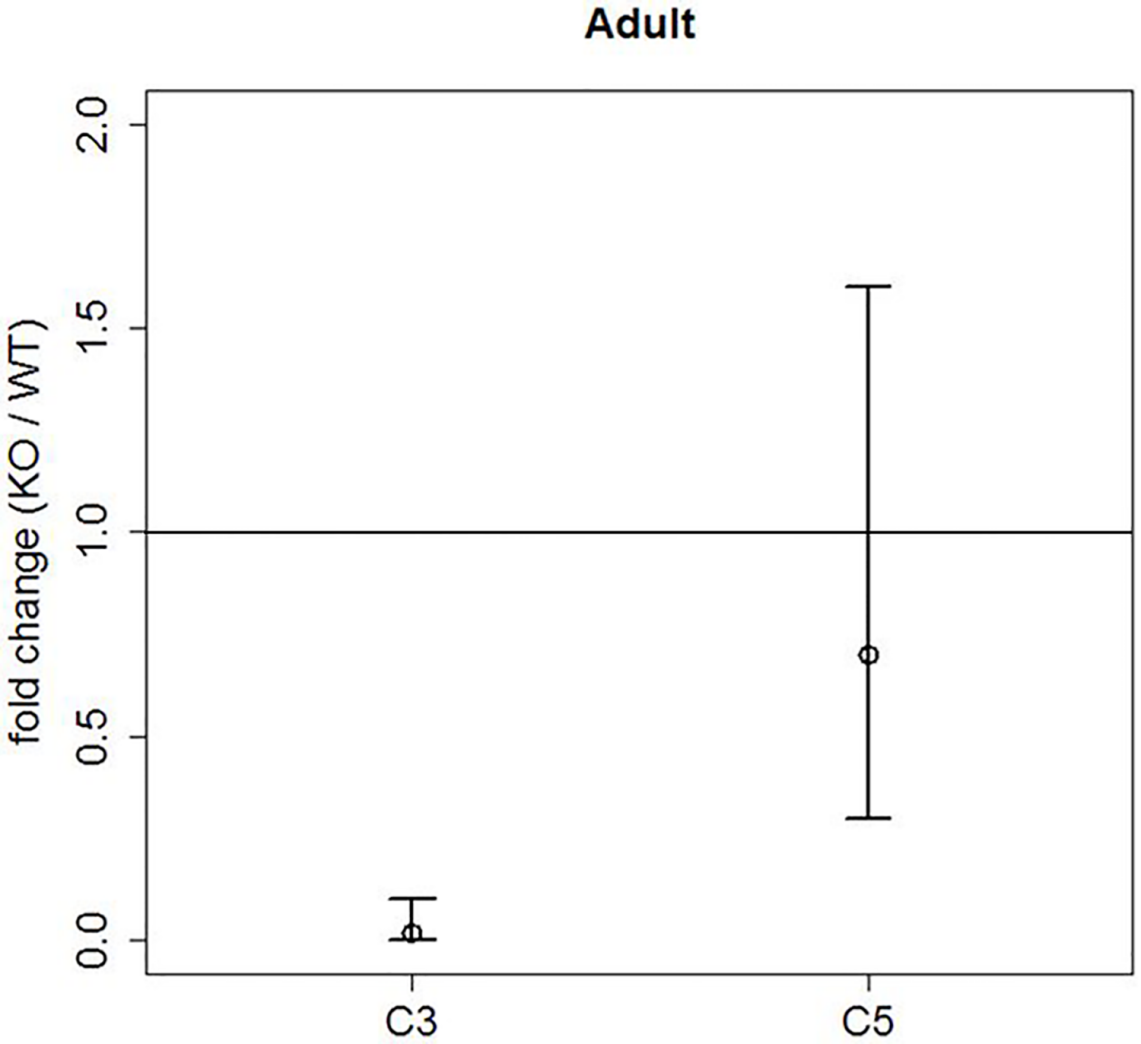
Graphic representation of the analysed qRT-PCR between adult WT and *SCCx43KO*^*-/-*^ mice. The horizontal line represents the null hypothesis (fold change equal to 1). C3: claudin-3; C5: claudin-5. The small circles denote the fold changes of the mRNA expression in *SCCx43KO*^*-/-*^ mice (claudin-3: 0.02, claudin-5: 0.7). The vertical lines denote the corresponding 95%-confidence interval. A 95%-confidence interval not containing the null hypothesis represents a statistically significant result (claudin-3). A 95%-confidence interval including the null hypothesis represents a statistically non-significant result (claudin-5).

An upregulation of claudin-11 mRNA has already been demonstrated in adult *SCCx43KO*^*-/-*^ mice [9]. In the present study downregulation of claudin-3 mRNA in adult *SCCx43KO*^*-/-*^mice could be identified (Fig 7). Claudin-3 mRNA expression was decreased 64-fold in *SCCx43KO*^*-/-*^ mice. This result was statistically significant with p-value <0.05 and the 95%-confidence interval not containing the null hypothesis fold change equal to 1. Claudin-5 mRNA expression showed a milder decrease (1.42-fold) which was not statistically significant: The p-value was >0.05 and the 95%-confidence interval contained the null hypothesis fold change equal to 1, which means that there was no difference in mRNA expression between both genotypes.

### Initial forming of the BTB in adolescent *SCCx43KO*^*-/-*^ mice

#### Claudin-11 IHC

Since initial formation of the BTB takes place between day 10 and 19 p.p. in rodents [25, 26], the IHC of the essential TJ protein claudin-11 was performed in adolescent WT and *SCCx43KO*^*-/-*^ mice at 2, 8, 12, 14, 16 and 23 days of age.

**Fig 8:**
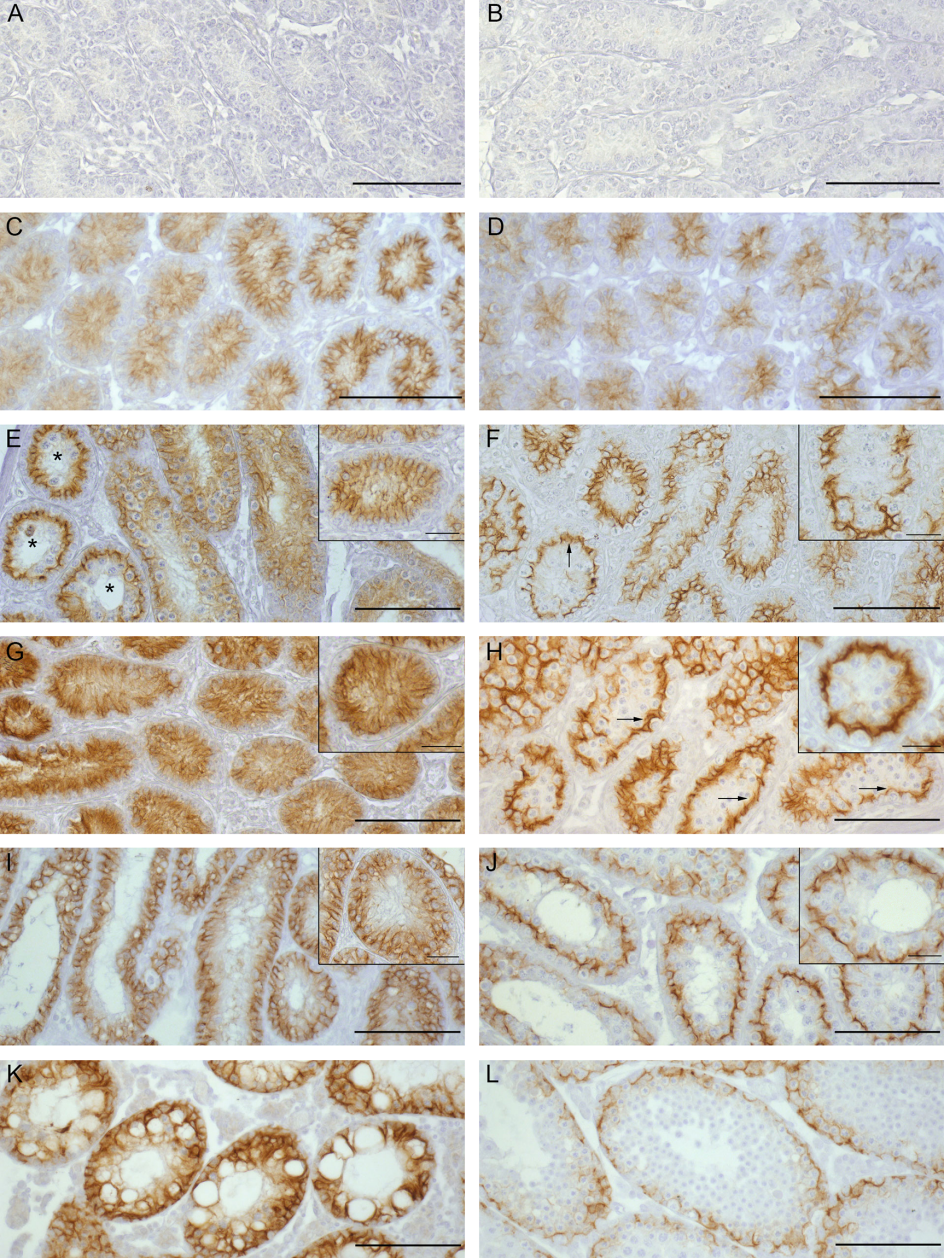
Claudin-11 immunohistochemistry in pre- and peripubertal WT and *SCCx43KO*^*-/-*^ mice. (A, C, E, G, I, K) Testes of *SCCx43KO*^*-/-*^ mice; (B, D, F, H, J, L) testes of WT mice. Postnatal development in days: Aged 2 (A and B), aged 8 (C and D), aged 12 (E and F), aged 14 (G and H), aged 16 (I and J) and aged 23 (K and L). (A and B) At the age of 2 days no claudin-11 protein is detectable in either of the genotypes. (C and D) A clear immunopositive but cytoplasmic reaction is visible at day 8 p.p. in both genotypes. (F, H, J, L) From day 12 p.p. a basal shift towards the BTB is visible in WT mice (arrows). (E (inset) G, I, K) In the seminiferous epithelium of *SCCx43KO*^*-/-*^ mice a cytoplasmic immunolocalisation for claudin-11 is observable over the whole-time period. (E, asterisks) Only in tubules with residual spermatogenesis could an age-dependent shift of claudin-11 towards the BTB be observed. From day 12 p.p. lumen formation occurs in both genotypes. Scale bars: 100 μm. Insets are showing one representative tubule with basal localisation of claudin-11 in WT and diffuse cytoplasmic localisation in *SCCx43KO*^*-/-*^ mice. Scale bars: 25 μm.

On day 2 p.p., no immunopositive reaction of claudin-11 could be seen in seminiferous cords of either *SCCx43KO*^*-/-*^ or WT mice (Fig 8A and 8B). On day 8 p.p., a clearly positive, but diffuse cytoplasmic immunostaining was visible in both genotypes (Fig 8C and 8D). Four days later, a basal shift of claudin-11 towards the BTB could be observed in some tubules in WT mice (Fig 8F, arrow and inset), on day 14 p.p., almost a quarter of the WT tubules showed a basal localisation of claudin-11 (Fig 8H, arrows and inset) and on day 16 p.p., already three quarters of the WT tubules displayed a basal localisation of claudin-11 immunostaining (Fig 8J). On day 23 p.p., WT tubules showed the same fine linear staining pattern in the basal part of the seminiferous epithelium as seen in adult WT mice (Figs 6A and 8L). In contrast, *SCCx43KO*^*-/-*^ mice showed a mostly diffuse distribution pattern of claudin-11 immunostaining in the SC cytoplasm over the whole investigation period (days 2–23 p.p., Fig 8A, 8C and 8E (inset), Fig 8G, 8I and 8K)). The immunostaining signal seemed to be stronger in *SCCx43KO*^*-/-*^ mice from day 14 onwards. Only in tubules with residual spermatogenesis could an age-dependent shift of immunolocalisation towards the BTB be observed in mutant mice (Fig 8E, asterisks). From day 12 p.p., lumen formation occurred in both genotypes and on day 23 all tubules had an open lumen.

#### Claudin-11 qRT-PCR

**Fig 9:**
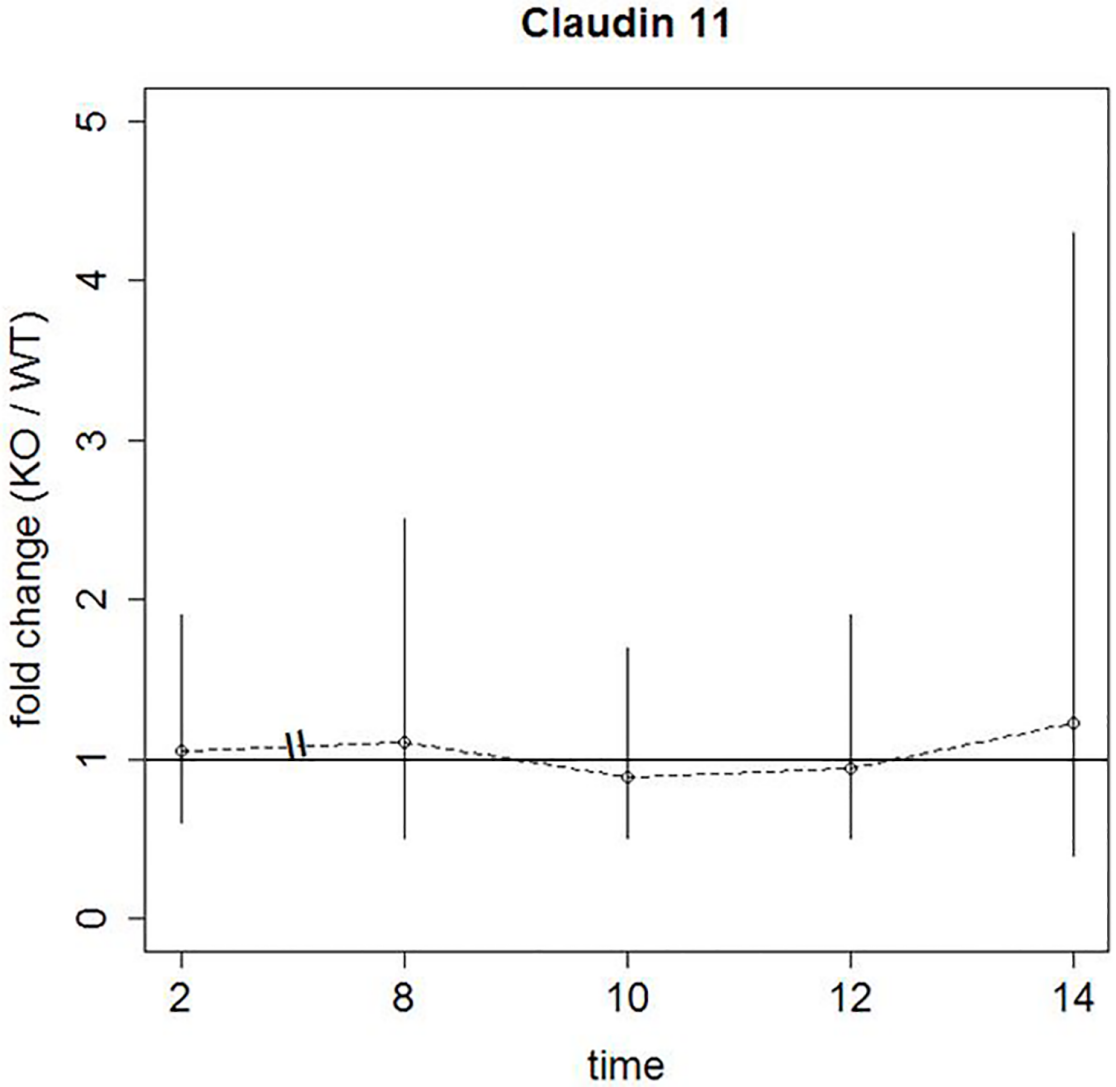
Graphic respresentation of the analysed qRT-PCR between adolescent WT and *SCCx43KO*^*-/-*^ mice. The horizontal line represents the null hypothesis (fold change equal to 1). The small circles denote the fold changes of the mRNA expression in *SCCx43KO*^*-/-*^ mice (day 2 p.p.: 1.05, day 8 p.p.: 1.11; day 10 p.p.: 0.89, day 12 p.p.: 0.94, day 14 p.p.: 1.23, respectively). The vertical lines denote the corresponding 95%-confidence interval. A 95%-confidence interval including the null hypothesis represents a statistically non-significant result.

It is already known that claudin-11 mRNA expression seems to be increased in adult *SCCx43KO*^*-/-*^ mice [9]. In the present study, mRNA expression of claudin-11 was additionally investigated in adolescent WT and *SCCx43KO*^*-/-*^ mice aged 2, 8, 10, 12 and 14 days p.p. There were no significant differences in mRNA levels between both genotypes at these ages (Fig 9).

For evaluation, n = 3 mice/genotype and age were analysed. At day 10 p.p., one of the *SCCx43KO*^*-/-*^ testis showed a deviating result and represented a statistical outlier. It was identified that the frozen testis of this animal had already been used in a former study to produce cryo-sections. In this context it might be possible that repeated freezing and thawing caused tissue quality changes which were responsible for the deviating result. For this reason, this testis was excluded from evaluation and on day 10 p.p. only n = 2 *SCCx43KO*^*-/-*^ mice were analysed.

#### Functional investigation

To determine the integrity of the BTB in peripubertal WT and *SCCx43KO*^*-/-*^ mice, hypertonic fixation was performed for the age groups 10, 12, 14, 16 and 23 days p.p.

At 10 days p.p., all tubules in both genotypes could be categorised as “open” because cell shrinkage was observed in/around all tubular cells. On day 12 p.p., both genotypes displayed some “intermediate” tubules as in these tubules shrinkage could be observed over several cell layers, but centrally located cells were already intact, indicating the formation of functional TJ which had not already arranged themselves uniformly in the basal third of the seminiferous epithelium. The proportion of intermediate tubules in *SCCx43KO*^*-/-*^ mice was almost three times as much than in WT mice. On day 14 p.p., first “closed” tubules could be found in both genotypes as evidenced by limitation of shrinkage artefacts to the lowest cell layer. The proportion of closed tubules rose during the investigation period in both genotypes but was always a bit higher in *SCCx43KO*^*-/-*^ mice. Data are shown in Table 4.

**Table 4:** Results of the functional investigation in adolescent WT and SCCx43KO^-/-^ mice.

Age (days)	Genotype	n	BTB closed %	BTB intermediate %	BTB open %
10	WT	296	0	0	100
	*SCCx43KO*^*-/-*^	244	0	0	100
12	WT	276	0	7.2	92.8
	*SCCx43KO*^*-/-*^	269	0	21.9	78.1
14	WT	346	37.9	24.3	37.9
	*SCCx43KO*^*-/-*^	342	46.2	29.2	24.6
16	WT	303	52.1	35.3	12.5
	*SCCx43KO*^*-/-*^	290	67.2	27.2	5.5
23	WT	288	86.8	12.5	0.7
	*SCCx43KO*^*-/-*^	313	95.8	3.8	0.3

Taken together, these analyses revealed that *SCCx43KO*^*-/-*^ mice (1) formed an intact BTB during puberty at the same time period as WT mice, which (2) seemed to be accelerated, compared to WT mice.

## Discussion

### Expression of the TJ proteins claudin-3, -5 and -11 in adult *SCCx43KO*^*-/-*^ mice

Although occludin was firstly identified as a component of TJ, claudin-11 seems to have a higher impact on the BTB integrity in mice. Furthermore, it seems to be very important or even essential for initial BTB formation [40, 42, 43, 62]. In addition to claudin-11, claudin-3 and -5 are also associated with murine BTB formation and due to different distribution patterns also different functions can be assumed for these three TJ proteins [63].

It has been shown in various studies that connexin based GJ exert a regulatory effect on different TJ proteins like occludin and claudins via, for example the common adapter protein ZO-1 or different signaling molecules [64–69]. Furthermore, overexpression of Cx43 is capable of resealing BTB and rebooting spermatogenesis up to round spermatids after toxicant-mediated barrier disruption in rats [70]. The *SCCx43KO* mouse model provides a unique tool for long term in vivo studies concerning Cx43 function in the testis and previous studies already provide some evidence for an altered BTB composition in these animals [9, 23]. Thus, the aim of the present study was to investigate closer the expression pattern of further testicular TJ proteins/claudins to ascertain a possible cause of impaired spermatogenesis observed in adult *SCCx43KO*^*-/-*^ mice. It is hypothesised that the spermatogenic disorders might be caused by an altered BTB assembly and/or disassembly, composition or regulation. For this purpose, not only claudin-11 but, for the first time, also the stage specific TJ proteins claudin-3 and -5 were investigated in tubules with and without residual spermatogenesis.

All three investigated claudins showed an altered (but different) expression pattern in adult *SCCx43KO*^*-/-*^ mice. In tubules with SCO syndrome and/or spermatogonial arrest, an apparently stronger immunoreaction as well as a diffuse cytoplasmic distribution pattern for claudin-11 in SC could be observed compared to WT mice. In contrast, claudin-3 und -5 could not be detected immunohistochemically in these tubules. Immunohistochemical data were confirmed at mRNA level using qRT-PCR. A statistically significant higher mRNA expression for claudin-11 was already demonstrated by Gerber et al. [9]. Claudin-3 mRNA expression was significantly decreased (64-fold) in the present study, whereas claudin-5 mRNA expression showed a lesser decrease (1.42-fold), which was not statistically significant. The low decrease in claudin-5 mRNA expression was at first unexpected because no claudin-5 protein was detectable in *SCCx43KO*^*-/-*^ tubules without residual spermatogenesis. However, in the testis, claudin-5 is not solely expressed in the seminiferous epithelium but also in endothelial cells [39]. In the present study, homogenates of whole testes were used in qRT-PCR, hence it was not possible to distinguish between claudin-5 expression in endothelial cells and the seminiferous epithelium. Observations from hematoxylin eosin staining as well as claudin-5 IHC indicated that *SCCx43KO*^*-/-*^ mice may form more interstitial blood vessels (data not shown), which might explain the (only) slight difference in claudin-5 mRNA expression between both genotypes. To compare claudin-5 mRNA expression in seminiferous tubules, laser microdissection following qRT-PCR should be performed.

It can be stated that claudin-3 and -5 are apparently not important for BTB integrity as SCx43KO^-/-^ mice form an intact BTB [23] despite the loss of both claudins. Due to their stage-specific expression pattern claudin-3 and -5 are associated with BTB dynamics [38, 39, 45–47] and their loss indicates impaired dynamics of the BTB, resulting in an arrest of spermatogenesis in adult *SCCx43KO*^*-/-*^ mice.

In this context, it is still unknown as to why adult *SCCx43KO*^*-/-*^ mice exhibit a close BTB despite the altered expression pattern of claudin-11. In the present study it was demonstrated for the first time that the higher expression of claudin-11 is accompanied by a diffuse cytoplasmic distribution pattern in SC. Claudins are integral membrane TJ proteins [63] and normally their cytoplasmic distribution pattern indicates that no functional TJ are formed [56, 71, 72]. There are two possible explanations for an intact BTB in our *SCCx43KO*^*-/-*^ mice: The first one might be that only the excessive claudin-11 protein is deposited in the SC cytoplasm and that there is also claudin-11 protein in the cell membrane forming intact TJ. This phenomenon was already observed by Li et al. [65]. The second explanation might be that it is not claudin-11 but another yet unknown TJ protein, which is accountable for the “tight” BTB. One potential candidate could be claudin-1 [63, 73, 74]. To verify the components forming the intact BTB in adult *SCCx43KO*^*-/-*^ mice, immunoelectron microscopy for claudin-11 and claudin-1 should be performed in further studies.

Interestingly, up to 5% of the *SCCx43KO*^*-/-*^ tubules show advanced GC populations, due to so far unknown reasons [5]. Beta-galactosidase and Cx43 IHC were performed to confirm successful gene deletion and loss of Cx43-based gap junctions. Under this aspect, it was of great interest to investigate TJ proteins in these single tubules to identify a potential Cx43-independent influence of specific GC populations on TJ expression, BTB formation and dynamics. A direct influence of GC on SC maturation and TJ expression has already been shown in various studies [48, 75–78]. Regarding claudin-11 expression, an inhibitory effect of postmeiotic GC was observed by Florin et al. [79] and it is stated that this mechanism contributes to GC migration towards the adluminal compartment. This regulatory effect of postmeiotic GC was also seen in the present study. In tubules with residual spermatogenesis containing round spermatids, claudin-11 IHC showed a finer staining pattern and a localisation of the immunostaining towards the BTB.

For claudin-5, regulation by GC was already shown by Morrow et al. [39] and these results corresponded to the findings in the present study where claudin-5 protein was also detectable only in tubules with residual spermatogenesis. In these tubules, immunolocalisation of claudin-5 protein was strongly associated with spermatocytes. As GC lack functional TJ [30], the question arises regarding the function of claudin-5 in these cells. It is known that TJ do not only have adhesive but also signaling function [80] and this signaling function might support GC differentiation and/or migration across the BTB. Claudin-5 can for example activate soluble-type pro-matrix metalloproteinase 2 (pro-MMP2) [39, 81] which might result in a disruption of junctional complexes in the seminiferous epithelium [82]. Thus, claudin-5 might be involved in BTB dynamics by supporting cyclic depletion of TJ strands between adjacent SC and the loss of claudin-5 may indicate impaired BTB dynamics resulting in an arrest of spermatogenesis There is also evidence of TJ regulating gene expression as well as cell proliferation and differentiation [80]. Therefore, it is possible that claudin-5 in spermatocytes acts as a regulator of spermatogenesis. Further studies are required to confirm these theories/ideas.

Like claudin-5, claudin-3 was also only detectable in tubules with residual spermatogenesis. In tubules containing spermatocytes or round spermatids a heterogeneous staining pattern with immunopositive and negative reactions was observable in the basal part of the seminiferous epithelium, apparently independent from the containing GC population. Such a heterogeneous immunostaining pattern for claudin-3 was also reported by Rondanino et al. [83] in fresh testicular tissues. A possible explanation for the seemingly heterogeneous immunostaining results in the present study could be that different GC (sub)populations were not definitely determined in the histological sections at hand. GC populations were differentiated only on the basis of morphological criteria via light microscopy. A precise differentiation of GC via special (immuno)staining techniques might be helpful to identify a certain GC population which may influence claudin-3 expression. Claudin-3 expression is regulated by androgens as a KO of the AR in SC leads to a loss of claudin-3 in the seminiferous epithelium [38, 55, 84]. Claudin-3 regulation via AR is also possible/understandable in *SCCx43KO*^*-/-*^ mice since AR expression is altered in these animals, too [53]. It has been shown in former studies that AR expression in SC depends on the presence of postmeiotic GC in the seminiferous epithelium [56, 76, 85]. Therefore, it is reasonable that claudin-3 protein is also detectable only in *SCCx43KO*^*-/-*^ tubules with residual spermatogenesis. Claudin-3 can be associated with the formation of new TJ during GC movement across the BTB and seems not to be crucial for BTB integrity as in claudin-3 KO mice BTB integrity remains unaffected [46, 84]. So, like claudin-5, the loss of claudin-3 protein in adult *SCCx43KO*^*-/-*^ mice might indicate an impaired dynamic in these mutants and an Cx43-independent regulation via AR seems to be capable to facilitate BTB dynamics.

Based on these findings in tubules with residual spermatogenesis, it could be assumed that GC exerted a Cx43-independent influence on TJ expression and BTB dynamics, either directly as for claudin-5 and -11 or indirectly via the AR as for claudin-3. These cells were apparently capable of compensating the loss of Cx43-based gap junctions in the seminiferous epithelium. Further investigations should focus on possible reasons for residual spermatogenesis in adult *SCCx43KO*^*-/-*^ mice. The knowledge about mechanisms of Cx43-independent spermatogenesis and BTB regulation would provide great benefit to the research field of male infertility.

### Initial forming of the BTB in adolescent *SCCx43KO*^*-/-*^ mice

Since an intact BTB could already be detected in adult *SCCx43KO*^*-/-*^mice [23], claudin-11 IHC and qRT-PCR as well as functional investigations were performed to determine and compare initial formation of a functional SC barrier in peripubertal WT and *SCCx43KO*^*-/-*^ mice. Initial formation of BTB normally takes place between day 10 and 19 p.p. in rodents [25, 26].

Claudin-11 protein was first detectable in SC cytoplasm on day 8 p.p. in both genotypes. These findings did not coincide with a former study by Mazaud-Guittot et al. [42] where claudin-11 protein was not detectable before day 13 p.p. However, this discrepancy might be due to methodical differences regarding the claudin-11 IHC ([42] and present study). Thus, the detection method used in the present study seemed to be more sensitive and therefore, claudin-11 protein could be detected a few days earlier. The results concerning the localisation of claudin-11 in the seminiferous epithelium of WT mice were comparable in both studies with a diffuse cytoplasmic distribution pattern at day 13 p.p. [42] and at day 8, 10 and 12 p.p. in the present study, respectively. A basal localisation of claudin-11 at the area of the BTB in WT mice was observed at day 20 p.p in both studies. In contrast, no basal localisation of claudin-11 immunostaining was observed in peripubertal *SCCx43KO*^*-/-*^ mice during the entire time period (day 2–23 p.p.). This observation would rather suggest that there is no BTB formation in peripubertal *SCCxKO43*^*-/-*^ mice. However, observed lumen formation as well as the results from present functional investigations indicated that there definitely is BTB formation in these mice. Using functional investigation this supposition was evidenced by limitation of hypertonically induced cell shrinkage to the basal compartment of the seminiferous epithelium [27, 54] from day 14 p.p. onwards (“closed” tubules). At this age point a basal shift of claudin-11 towards the BTB could be observed in some tubules in WT mice. The proportion of closed tubules in the functional investigation rose constantly during postnatal/peripubertal development and in each age group there was always a higher proportion of these tubules in *SCCx43KO*^*-/-*^ mice. These first findings demonstrated BTB formation during puberty in *SCCx43KO*^*-/-*^mice, which however seemed to be accelerated. So, as described above for adult *SCCx43KO*^*-/-*^ mice, intact TJ are formed despite the diffuse cytoplasmic distribution pattern of claudin-11 protein. It may therefore be concluded that initial formation of functional TJ works in an Cx43 and GC independent manner as both stimuli are absent in *SCCx43KO*^*-/-*^ mice.

There were no significant differences in claudin-11 mRNA expression between WT and *SCCx43KO*^*-/-*^ mice between days 2–14 p.p. ([8] and present study). Claudin-11 mRNA is already expressed in fetal testes. After birth, expression starts to increase from day 3 p.p. onwards and reaches a plateau between day 6 and 16 p.p. From day 16 p.p., a decline in claudin-11 mRNA expression is detectable [41]. It may be possible that SC in *SCCx43KO*^*-/-*^ mice do not show this decline in mRNA expression and a difference becomes obvious from day 16 p.p. onwards. Thus, further age groups (e.g. 16–30 days old) should be examined to determine the precise starting point of a higher mRNA expression in adolescent *SCCx43KO*^*-/-*^ mice. Previous studies already provided some evidence that Cx43 might work as a down-regulator of tight and adherens junctions [9, 23]. The data of the present study support this hypothesis and demonstrate that Cx43 influences down-regulation of TJ rather than initial formation. Overexpression of different junction proteins, like claudin-11 ([9] and present study), occludin, ß-catenin and N-cadherin [23], results in a tighter BTB with impaired restructuring. BTB dynamics seem mainly be influenced by different GC populations as shown by claudin expression in tubules with residual spermatogenesis. But as it seems unlikely that spermatogenesis and BTB dynamics work completely GJ independent, further studies should be conducted regarding possible compensating mechanisms of Cx43 loss in tubules with residual spermatogenesis.

In conclusion, the findings of the present study provide evidence of impaired BTB dynamics in *SCCx43KO*^*-/-*^ mice indicated by (1) the absence of claudin-3 and -5 and (2) the increase of claudin-11 protein in the seminiferous epithelium. Claudin-3 and -5 seem apparently not to be important for the integrity of this barrier as adult mutants form an intact BTB despite the loss of these two claudins. Claudin-11 might form intact TJ despite its diffuse cytoplasmic distribution pattern or even another (so far unknown) TJ protein is responsible for TJ forming. Finally, it was shown for the first time that adolescent *SCCx43KO*^*-/-*^ mice formed a functionally intact BTB during puberty. The conditional *SCCx43KO*^*-/-*^ mouse model offers optimum conditions for further in vivo investigations on the regulation and formation of the BTB and can therefore contribute to a better understanding of the mechanistic roles of Cx43 in spermatogenesis and BTB assembly/disassembly. Especially *SCCx43KO*^*-/-*^ mice with residual spermatogenesis are suitable for these investigations as these animals show seminiferous tubules with and without spermatogenesis in close proximity.
